# Expression of the Antioxidative Enzyme Peroxiredoxin 2 in Multiple Sclerosis Lesions in Relation to Inflammation

**DOI:** 10.3390/ijms18040760

**Published:** 2017-04-04

**Authors:** David Voigt, Uta Scheidt, Tobias Derfuss, Wolfgang Brück, Andreas Junker

**Affiliations:** 1 Institute of Neuropathology, University Medical Center, Robert-Koch-Straße 40, Göttingen 37075, Germany; david-voigt@gmx.net (D.V.); uscheidt@med.uni-goettingen.de (U.S.); wbrueck@med.uni-goettingen.de (W.B.); 2 Neurologic Clinic and Policlinic, University Hospital Basel, Basel 4031, Switzerland; tobias.derfuss@usb.ch; 3 Institute of Neuropathology, University Hospital Essen, Hufelandstr. 55, Essen 45122, Germany

**Keywords:** multiple sclerosis (MS), peroxiredoxin 2 (PRDX2), NAD(P)H quinone dehydrogenase 1 (NQO1), oxidative stress, inflammation

## Abstract

Multiple sclerosis is a chronic inflammatory disease of the central nervous system, characterized by demyelination and axonal damage as well as neuronal degeneration. Since oxygen-derived free radicals are an important factor leading to tissue damage in inflammatory multiple sclerosis (MS) lesions, research on antioxidative systems is essential to identify endogenous factors which can possibly counteract oxidative damage. As an important scavenging enzyme family, peroxiredoxins (PRDXs) play a crucial role in preventing oxidative damage; however little is known about their expression and function in MS lesions. In the present study we examined the expression of PRDX2 in white matter lesions of MS patients with long-standing, chronic disease. PRDX2 expression was investigated by immunohistochemistry in the context of oxidative stress and inflammation (determined by microglia/macrophage and T cell infiltration) in ten MS autopsy cases as well as seven control autopsy cases. PRDX2 was found to be upregulated in white matter MS lesions mainly in astrocytes, and its expression level was positively correlated with the degree of inflammation and oxidative stress. Our data suggest that PRDX2 expression contributes to the resistance of astrocytes against oxidative damage.

## 1. Introduction

Multiple sclerosis (MS) is a chronic inflammatory, demyelinating disease of the central nervous system (CNS) and is associated with axonal damage and neuronal degeneration. The pathomechanisms of tissue damage have not yet been fully elucidated. One important mechanism contributing to demyelination and neurodegeneration is the accumulation of reactive oxygen species (ROS) and reactive nitrogen species (RNS) in inflammatory MS lesions [1,2]. In general, oxygen-derived free radicals play an important role during inflammation and cell aging, and have also been described in other neurodegenerative diseases, such as Alzheimer’s disease [3], Parkinson’s disease [4], Huntington’s chorea [5] and amyotrophic lateral sclerosis [6].

At the molecular level ROS and RNS alter proteins (oxidation), polyunsaturated fatty acids in membrane lipids (peroxidation) and damage DNA/RNA (strand breaks). However, cells of the CNS are able to protect themselves against ROS and RNS by inactivating these highly reactive molecules and by repairing emerging damage [7]. This capability critically depends on the expression of antioxidative molecules which is regulated e.g., through activation of the transcription factor nuclear factor (erythroid-derived 2)-like 2 (Nrf2) [8]. Activated Nrf2 alters the expression of several proteins, such as NAD(P)H quinone dehydrogenase 1 (NQO1). Therefore, alterations in NQO1 expression were proposed as a marker for cellular oxidative stress [9].

As an important family of antioxidative proteins, peroxiredoxins were only sporadically identified in MS lesions in the past [10,11]. Peroxiredoxins are ubiquitously expressed throughout prokaryotes and vertebrates [12,13] and six different peroxiredoxins are known in humans [14]. One of these is peroxiredoxine 2 (PRDX2), which is highly abundant in human cells and displays a high reaction rate with hydrogen peroxide as shown in various kinetic studies [15,16,17]. In its reduced state PRDX2 catalyzes the reduction of peroxides (R–OOH) to alcohols (R–OH) while being oxidized at a cysteine residue, and in a second step it is reconstituted via the oxidation of a thiol, frequently thioredoxin [14]. Interestingly, it not only acts as a scavenging enzyme and prevents oxidative damage, but it is also a modulator of intracellular redox signaling [18,19]. Furthermore, extracellular release of PRDX2 from necrotic brain cells is an initiator of post-ischemic inflammation in the brain through activation of Toll-like receptors [20].

The cytoprotective antioxidative activity of PRDX2, its modulatory role in intracellular redox signaling, and its function as a potent extracellular activator of Toll-like receptors make PRDX2 an interesting molecule to investigate in the context of inflammatory demyelinating diseases, such as MS, in which oxidative stress is described as a mechanism leading to demyelination, axonal damage and neurodegeneration.

The objective of our study was to systematically investigate the expression of PRDX2 in chronic white matter MS lesions as well as in normal appearing white matter (NAWM) of patients with and without MS (controls). We found that PRDX2 was mainly expressed in astrocytes, and its expression was associated with oxidative stress and inflammation (microglial/macrophage activation, T cell infiltration).

## 2. Results

### 2.1. Tissue and Lesion Classification

All tissue blocks underwent detailed neuropathological examination (Andreas Junker and Wolfgang Brück) and were screened for grey and white matter myelin damage. For a detailed evaluation of the lesions we performed immunohistochemistry using antibodies against proteolipid protein (PLP) and myelin basic protein (MBP) to visualize myelin sheaths. In order to quantify and characterize the inflammatory infiltrate, we used anti-CD3 antibodies as a marker for T cells and the anti-CD68 equivalent antibody clone KiM1P as a marker for microglia/macrophages. Tissue samples from ten MS patients as well as from seven patients without neurological disease (controls) were used in the study. Four white matter lesions from three different MS patients showed an enhanced number of T cells and abundant macrophages, which contained myelin degradation products at the lesion edge. They were therefore classified as chronic active plaques (CAP). Five white matter lesions from three other MS patients showed only little macrophage or T cell infiltration and were considered to be chronic inactive plaques (CIP). Four cases of MS without lesions in the observed material were evaluated.

### 2.2. Astrocytes Show Enhanced Expression of PRDX2 in WML

**Figure 1:**
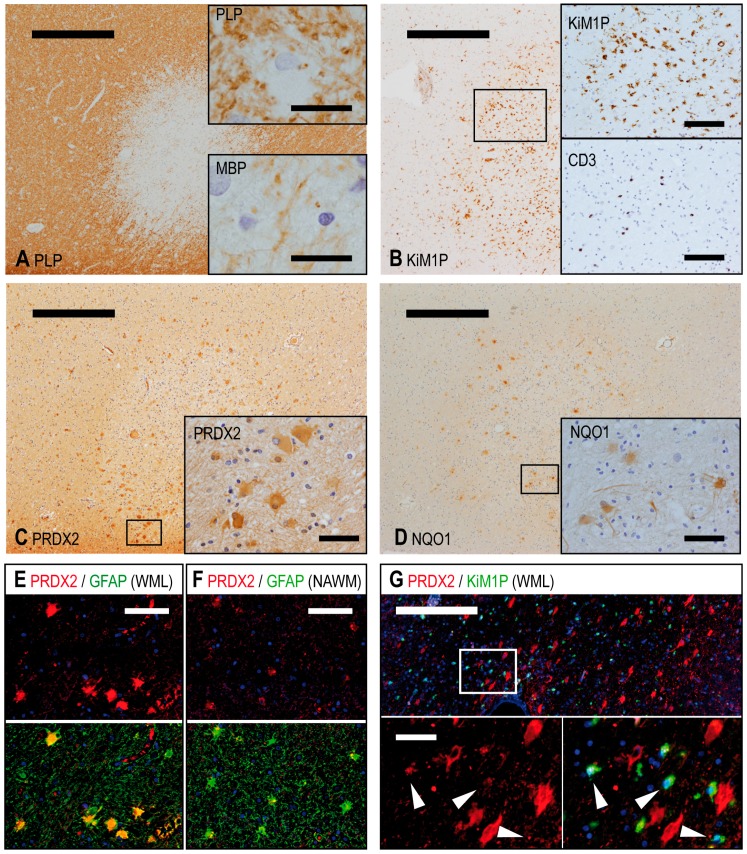
Chronic active white matter lesion (WML) with abundant astrocytic expression of PRDX2. Demyelination was detected by PLP immunohistochemistry (**A**). A few macrophages at the lesion edge contained myelin degradation products, which stained positive for MBP (**A**, Insert) and PLP (**A**, Insert). Microglia/macrophages were identified by immunohistochemical staining for KiM1P/CD68 (**B**) and T cells for CD3 (**B**, Insert). A high expression of PRDX2 (**C**) and NQO1 (**D**) was seen in the same lesion. Double immunofluorescence staining with GFAP confirmed that PRDX2 was mainly expressed by astrocytes (**E**, further pictures are provided in the Figure S1). Immunofluorescence staining of an additional white matter lesion exhibited a weak expression of PRDX2 (**G**, arrows) in KiM1P-positive macrophages. Scale bars: **A**–**D**: 500 µm, **A** Inserts: 20 µm, **B** Inserts: 100 µm, **C**–**D** Inserts: 50 µm, **E**–**F**: 30 µm, **G**: 200 µm, **G** Inserts: 30 µm.

To characterize the expression of PRDX2, all tissue samples were stained immunohistochemically for PRDX2. In the white matter, PRDX2 was predominantly expressed in the cytoplasma of CNS resident cells, which had a star-shaped appearance (multipolar, branching cytoplasmatic processes) and a round nucleus. Double immunofluorescence staining with glial fibrillary acid protein (GFAP) confirmed that these cells were astrocytes. The greatest number of astrocytes expressing PRDX2 was found at the edge of white matter lesions (WML) of MS patients (Figure 1). PRDX2 expression was found to a lesser extent in astrocytes of normal appearing white matter (NAWM) in MS as well as in control material. Microglia/macrophages were identified by double immunofluorescence staining using the antibody KiM1P. They showed a slight cytoplasmatic and more granular PRDX2 positivity in WML.

With regard to grey matter, the most striking PRDX2 expression was found in neurons in leukocortical lesions (Figure S2), in which the adjacent white matter showed an accumulation of inflammatory cells.

### 2.3. PRDX2 Expression Correlates with Inflammation and Oxidative Stress in WML

**Figure 2:**
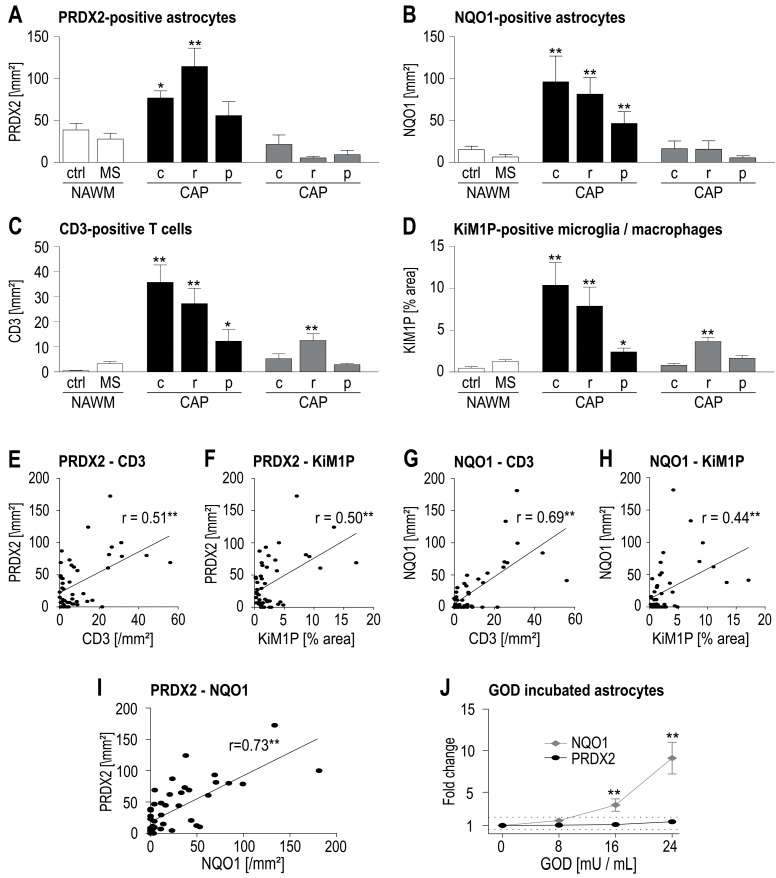
PRDX2 and NQO1 expression (**A**,**B**) in relation to inflammation (**C**–**H**) and oxidative stress (**I**,**J**). PRDX2-/NQO1-expressing astrocytes, T cells (CD3) and activation of microglia/macrophages (KiM1P) were evaluated in the normal appearing white matter (NAWM) of controls (ctrl) and MS patients as well as in four chronic active MS plaques (CAP) and five chronic inactive MS plaques (CIP). All measurements were performed separately in the center of the lesions (c), the rim of the lesions (r) and the peri-plaque white matter (p). Within the lesions, a comparable distribution pattern of CD3, KiM1P as well as astrocytic PRDX2 and NQO1 expression was found (**A**–**D**). The highest expression of these markers was observed in the center and at the rim of CAP, and was significantly higher than in MS NAWM or in white matter of controls (Mann–Whitney *U*-test). The number of PRDX2- and NQO1-expressing astrocytes showed a significant positive correlation (Pearson r) with the number of T cells and the activation of microglia/macrophages (PRDX2 in (**E**,**F**), NQO1 in (**G**,**H**)). Moreover, a strong positive correlation between PRDX2 and NQO1 expression was found (**I**). In vitro experiments showed that incubation of primary murine astrocytes with glucose oxidase (GOD) for 24 h increased NQO1 but not PRDX2 in the cells as measured by quantitative PCR ((**J**), ANOVA, error bars represent SD). ** *p* < 0.01, * *p* < 0.05.

Since we observed the greatest number of PRDX2-expressing astrocytes in WML of MS patients, we investigated to which extent PRDX2 expression was correlated with inflammation. With regard to the architecture of chronic MS lesions, all measurements were performed separately in the center and at the rim of the lesions as well as in the peri-plaque white matter. As expected, the greatest number of CD3-positive T cells and a remarkable activation of microglia/infiltration of macrophages (stained with the antibody KiM1P) were found in the center and at the rim of chronic active plaques (CAP) (Figure 2A,B). However, the greatest number of PRDX2-positive astrocytes was found at the rim of CAP (Figure 2C). Combining the measurements from all investigated regions (lesion center, lesion rim, peri-plaque white matter, NAWM of MS and control cases) a weak, albeit significant, positive correlation was found between the number of PRDX2-expressing astrocytes and the T cell infiltrate (Figure 2D) as well as the activation of microglia/macrophages (Figure 2E).

Unlike inflammatory cells, oxygen-derived free radicals cannot be directly detected in autopsy tissue. High levels of oxidative stress, as observed in acute inflammatory MS lesions, can be indirectly inferred by measuring oxidized phospholipids, proteins or DNA [1,2], which are not detectable in chronic MS lesions (own observation). Since oxygen-derived free radicals influence the expression of several proteins by activating the transcription factor Nrf2, these molecules may serve as markers for chronic low-level oxidative stress. NQO1, a downstream Nrf2 regulated protein, was described previously in chronic MS lesions and is mainly expressed by astrocytes [9]. Based on these findings we used NQO1 as a marker for oxidative stress in our study. In MS patients NQO1 expression was mainly observed in astrocytes in the center and at the rim of CAP (Figure 1D and Figure 2F). The distribution of NQO1-expressing astrocytes in the investigated WML largely resembles the distribution pattern of activated microglia/macrophages and T cell infiltration. We found a positive correlation between the number of NQO1-positive astrocytes and the number of infiltrating T cells (Figure 2G). Moreover, a striking positive correlation was found between the numbers of NQO1- and PRDX2-expressing astrocytes.

In summary, we found a co-localization of inflammation and astrocytic PRDX2 and NQO1 expression. Since inflammatory cells are not only a potent source of oxygen-derived free radicals but also produce cytokines, we investigated to what extent oxidative stress in itself triggers the expression of PRDX2 and NQO1, while also addressing the question whether astrocytic NQO1 is a reliable marker for oxidative stress. In vitro we found that murine astrocytes incubated with glucose oxidase (GOD), an enzyme that catalyzes the synthesis of hydrogen peroxide, showed enhanced NQO1 expression at the RNA level in a concentration-dependent manner (Figure 2J). However, no enhanced PRDX2 expression was observed at the transcriptional level after incubation of astrocytes with GOD. Similar results were found in the human neuroblastoma cell line SH SY5Y (material not intended for publication). Our data suggest that astrocytic NQO1 expression can indeed be used as a marker for oxidative stress. Further studies must show which factors lead to enhanced PRDX2 expression in astrocytes. We did not find enhanced transcription of PRDX2 or NQO1 after incubation of murine astrocytes with IL-1β or IFN-γ (material not intended for publication), cytokines that are known to activate astrocytes [21,22].

## 3. Discussion

In the present study we characterized for the first time the expression of the antioxidative enzyme PRDX2 in CNS autopsy tissue of patients with MS and patients without reported or neuropathologically detectable brain diseases (controls). Since oxidative stress is considered to be a tissue damaging mechanism in MS [1,2,23], we focused on cell protective antioxidative molecules in our study. In addition to glutathione peroxidase, catalase and other extensively studied antioxidative molecules, the family of peroxiredoxins participates substantially in the detoxification of free radicals [24]. PRDX2 is an important member of this enzyme family due to its great abundance in human cells and its high reaction rate with hydrogen peroxide [17]. Its cytoprotective function was also demonstrated in PRDX2 knockout mice, in which the loss of PRDX2 resulted in hemolytic anemia [25]. This is an interesting finding because erythrocytes are chronically exposed to oxidative stress under physiological conditions. Further studies will have to analyze the effects of PRDX2 loss under pathophysiological conditions such as experimental autoimmune encephalomyelitis, an animal model for MS. Besides its cytoprotective antioxidative activity, PRDX2 also modulates intracellular redox signaling [18] and is a potent extracellular activator of Toll-like receptors [20,26].

Here, we showed that PRDX2 is predominantly expressed by astrocytes in the white matter. The highest number of PRDX2-expressing astrocytes was found at the rim of chronic active MS plaques (CAP). A slight expression was also observed in activated microglia/macrophages. Our data support the view that different cell types in the CNS vary in their ability to cope with oxidative stress. In MS, a disease which is characterized by demyelination and neuroaxonal degeneration, apoptotic oligodendrocytes and transected neurites were found to be associated with oxidative stress [2]. In active white matter lesions of MS patients the majority of cells with oxidized DNA and oxidized phospholipids are oligodendrocytes [2]. Astrocytes and microglia/macrophages are discussed as potent sources of oxygen-derived free radicals [1,11] and seem to be less damaged by their own products.

Furthermore, we found a positive correlation between PRDX2 expression in the astrocytes and the local inflammatory infiltrate (T cells and activated microglia/macrophages). Microglia/macrophages are a potent source of oxygen-derived free radicals [11]. But T cells, microglia/macrophages, and astrocytes also communicate through the release of cytokines [27]. To investigate which factors lead to an enhanced astrocytic PRDX2 expression, we first focused on oxidative stress. As a marker for chronic low-level oxidative stress we used the Nrf2-regulated molecule NQO1 [9], which showed, as does PRDX2, an enhanced expression in astrocytes in relation to the local inflammatory infiltrate. In addition, we found a strong positive correlation between the local number of PRDX2- and NQO1-expressing astrocytes. This observation might imply that astrocytes protect themselves against oxidative stress by increasing PRDX2 expression in regions of increased oxidative stress.

Still, the question remained which factors regulate PRDX2 expression, and whether NQO1 is an indicator for oxidative stress or is, rather, upregulated upon activation of astrocytes by cytokines. In vitro we found enhanced NQO1 expression at the transcription level in murine astrocytes after incubation with glucose oxidase (GOD), indicating that NQO1 expression is, indeed, a marker for oxidative stress. No altered NQO1 expression was found after incubation of astrocytes with IL-1β or IFN-γ (material not intended for publication). However, neither incubation with GOD nor with IL-1β or IFN-γ (material not intended for publication) resulted in an altered transcription of PRDX2.

In summary, our data suggest that NQO1 is a marker for low-level oxidative stress, that astrocytic PRDX2 expression is enhanced in brain regions with increased levels of oxygen-derived free radicals in MS, and that PRDX2 expression is not directly influenced by the presence of oxidative stress. Thus, we assume that PRDX2 expression is regulated by cytokines, although we did not find evidence of upregulation after incubation of astrocytes with IL-1β or IFN-γ at the RNA level. If oxidative stress, IL-1β or IFN-γ influenced PRDX2 expression, it might be on a posttranscriptional level.

In conclusion, we present a detailed expression profile of PRDX2 in the white matter, a molecule with a potentially key role in detoxifying oxygen-derived free radicals in inflammatory MS lesions. We showed that PRDX2 expression varied between different CNS resident cells. This could at least partially explain their different vulnerability towards oxidative stress. A better understanding of the regulation of detoxifying proteins might help to develop therapeutic strategies for tissue protection.

## 4. Material and Methods

### 4.1. Human Brain Tissue

This study was performed on formalin-fixed, paraffin-embedded (FFPE) archival brain tissue from ten autopsy MS cases as well as seven age and region matched controls (Table 1 and Table S1). All patients suffered from long-lasting chronic multiple sclerosis.

MS and control cases were diagnosed at the Institute of Neuropathology, University Medical Center Göttingen, Germany. Tissue blocks were obtained from the frontal, temporal or occipital lobe. Two neuropathologists (Andreas Junker and Wolfgang Brück) confirmed the neuropathological diagnosis of all patients. In addition, whenever available, the clinical history and the results of whole body autopsy were reviewed. All investigations were performed in compliance with relevant laws and institutional guidelines, and were approved by the local ethics committee. The investigations were carried out following the rules of the Declaration of Helsinki of 1975, revised in 2008.

### 4.2. Histology and Immunohistochemistry

All staining procedures were performed on 3 µm thick tissue sections. In order to assess basic pathologic changes and demyelination, a section of each tissue sample was stained with hematoxylin-eosin (HE) as well as with Luxol fast blue (LFB) myelin stain in combination with periodic acid-Schiff (PAS) reaction. Axons were stained with Bielschowsky’s silver impregnation. For immunohistochemistry, sections were pretreated as listed in Table S2. Endogenous peroxidase activity was blocked by incubation of the sections in 3% H_2_O_2_ in PBS. To prevent unspecific antibody binding, sections were placed in 10% fetal calf serum in PBS for 10 minutes at room temperature. Subsequently, sections were incubated with the primary antibodies overnight, followed by the corresponding secondary antibodies, which were either fluorescence- or biotin-labeled. Biotin-labeled antibodies were visualized using an avidin-biotin technique with 3,3′-diaminobenzidine (DAB) as chromogen. Nuclei were stained with hematoxylin for light microscopy or with 4′,6-diamidino-2-phenylindole (DAPI) for fluorescence microscopy. The antibody concentrations and pretreatments used are listed in supplementary Table S2.

### 4.3. Morphometric Analysis and Data Acquisition

PRDX2- and NQO1-positive astrocytes were counted manually in 5–7 digital photographs per region of interest at 200× magnification (image size 0.144 mm^2^). The number of T cells (stained for CD3) was evaluated using an ocular counting grid (Olympus WHN 10X/22) at 200× magnification (field size 0.25 mm^2^). T cells in eight fields per region of interest were counted manually. Microglia/macrophages were stained immunohistochemically with the antibody clone KiM1P and 5–7 pictures in each region of interest were taken at 200× magnification. As a readout for the activation state of microglia/macrophages, the KiM1P positive area of each picture was measured and calculated in percent by using the ImageJ functions “color threshold” and “analyze particles” [28].

### 4.4. Cell Culture Experiments with Primary Murine Astrocytes

C57B6/J mice were purchased from the Charles River laboratory (San Diego, CA, USA). Astrocytes were isolated as described previously [29]. Cells were cultured in DMEM supplemented with 10% fetal calf serum (Gibco, Life Technologies GmbH, Darmstadt, Germany), 100 U/mL penicillin and 0.1 mg/mL streptomycin (Sigma-Aldrich Chemie GmbH, Steinheim, Germany) at a temperature of 37 °C in 5% CO_2_. For each single experiment 100,000 cells were used per well. To induce oxidative stress, cells were incubated with glucose oxidase (GOD, Sigma-Aldrich Chemie GmbH) for 24 h in concentrations up to 24 mU/mL. Experiments were performed in triplicate. All animal procedures used in this study were in accordance with the guidelines of the committee on animals of the University Medical Center Göttingen with the permission of the Regierung von Niedersachsen (Hannover, Germany).

### 4.5. RNA Extraction and cDNA Synthesis

RNA was isolated from cell cultures using the miRNeasy mini-kit (Qiagen, Hilden, Germany) according to the protocol described by the manufacturer. To obtain a higher yield of RNA, we used RNeasy micro-spin columns (Qiagen). RNA concentration was determined with the NanoDrop (Peqlab Biotechnologie GmbH, Erlangen, Germany). Isolated RNA was stored at −80 °C.

Trancription of RNA into complementary DNA (cDNA) was performed with the High-Capacity cDNA Reverse Transcription Kit (Life Technologies GmbH) according to the manufacturer‘s instructions. For each reaction (20 µL) 200 ng RNA were used.

### 4.6. Quantitative PCR

The qPCR reactions were performed using the qPCR Core Kit and uracyl *N*‑glycosylase (both from Eurogentec, Cologne, Germany). Transcripts were detected with TaqMan Assays (Applied Biosystems, Darmstadt, Germany) for βActin (Mm00607939_s1), NQO1 (Mm0125356_m1) and PRDX2 (Mm04208313_g1). A total amount of 10 ng cDNA was used for each reaction (20 µL). Δ*C*_t_ was calculated as difference between the *C*_t_ of the gene of interest (NQO1 or PRDX2) and the *C*_t_ of the used housekeeping gene (βActin). To calculate the ΔΔ*C*_t_, the Δ*C*_t_ of untreated cells was subtracted from the Δ*C*_t_ of GOD treated cells.

### 4.7. Statistical Analysis

Statistical anaylsis was performed with GraphPad Prism 5.0. The Mann-Whitney *U*-test was used to compare independent groups with non-parametric data. The correlation between groups was calculated as Pearson r. One-way ANOVA for parametric data was used for comparing more than two groups. A *p*-value of <0.05 * (<0.01 **) was considered statistically significant.

## 5. Conclusions

We showed that the antioxidative enzyme PRDX2 is upregulated in white matter MS lesions mainly in astrocytes, and its expression level is positively correlated with the degree of inflammation and oxidative stress. Our data suggest that PRDX2 expression contributes to the resistance of astrocytes against oxidative damage.

## Figures and Tables

**Table 1:** MS autopsy cases and controls.

Case	Age	Sex	Cause of Death	Inflammatory Circumstances Perimortal
MS-01	74	female	pneumonia	pneumonia
MS-02	63	male	unknown	unknown
MS-03	57	male	pneumonia	sepsis
MS-04	51	female	circulatory collapse	no
MS-05	49	male	unknown	unknown
MS-06	49	male	drug abuse	no
MS-07	47	female	unknown	unknown
MS-08	44	female	unknown	unknown
MS-09	41	male	pulmonary embolism	no
MS-10	34	male	seizure	unknown
CON-01	67	female	adult respiratory distress syndrome	pneumonia
CON-02	63	female	cardiovascular failure	no
CON-03	56	male	lung cancer	unknown
CON-04	49	male	multiple organ failure	sepsis
CON-05	48	female	cardiovascular failure	no
CON-06	40	female	cardiovascular failure	no
CON-07	35	female	pulmonary embolism	no

## Supplementary Materials

Supplementary materials can be found at www.mdpi.com/1422-0067/18/4/760/s1.
