# Supplementary material for: Expression of the Antioxidative Enzyme Peroxiredoxin 2 in Multiple Sclerosis Lesions in Relation to Inflammation
Source: Int J Mol Sci. 2017 Apr 4;18(4):760. doi: 10.3390/ijms18040760 (PMC5412345; doi:10.3390/ijms18040760)
Supplement: Supplementary file 1 [file ijms-18-00760-s001.pdf]

Supplementary Material: Expression of the Antioxidative Enzyme Peroxiredoxin 2 in Multiple Sclerosis Lesions in Relation to Inflammation

David Voigt, Uta Scheidt, Tobias Derfuss, Wolfgang Brück and Andreas Junker

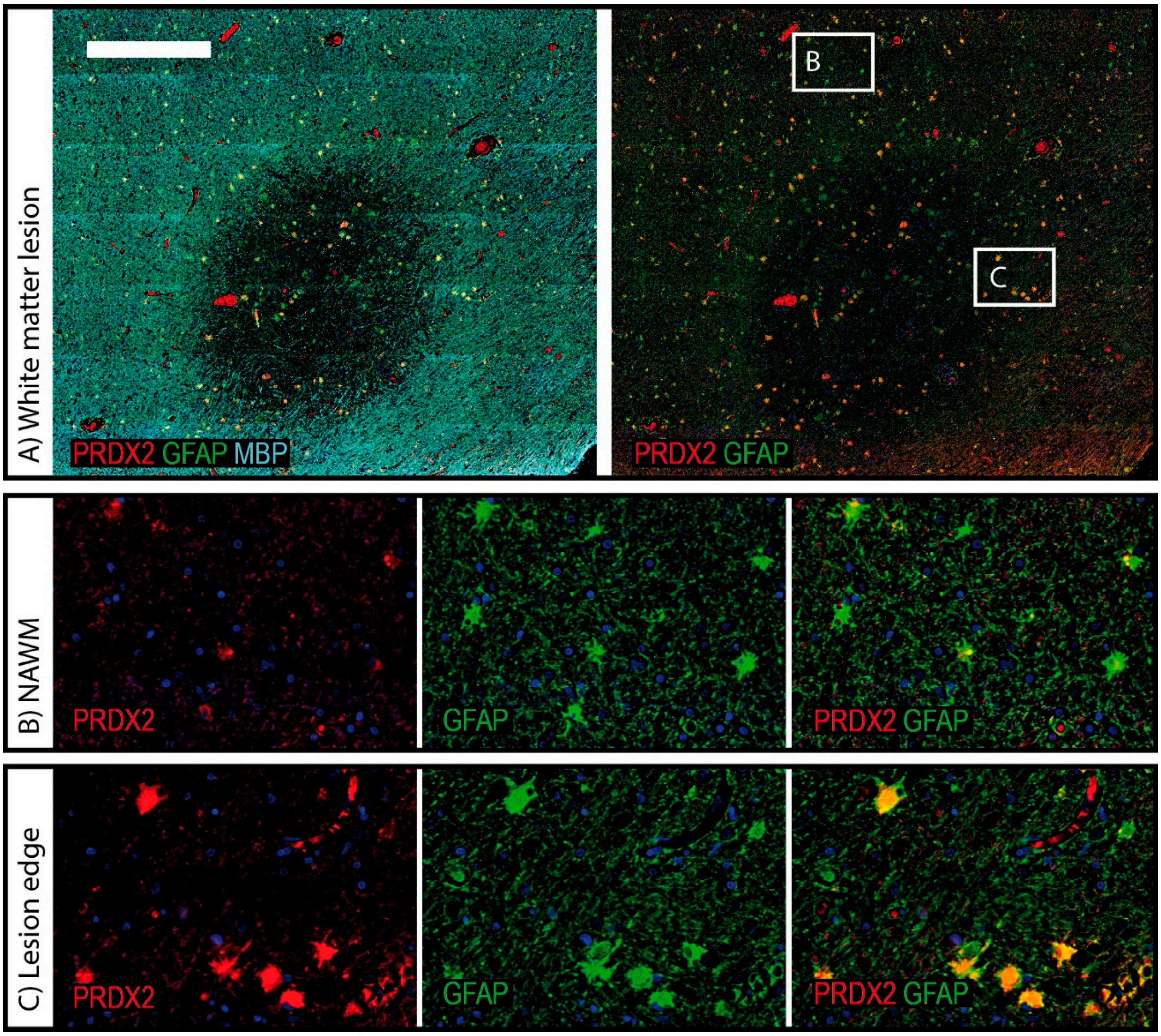

Figure S1. The same chronic active white matter lesion (WML) as shown in Figure 1, used here for a more detailed illustration of the PRDX2 expression in astrocytes (stained for GFAP). An overview of the lesion was given at low magnification in a triple fluorescence staining for PRDX2 (red), for the astrocytic intermediate filament GFAP (green) and the myelin protein MBP (cyan) (A). The marked areas in A were shown at a higher magnification in B and C. These pictures, except the green channel (GFAP) alone, were already used for Figure 1. While there were only a few astrocytes with a mild expression of PRDX2 in the normal appearing white matter (NAWM, B), astrocytic PRDX2 expression was more prominent at the lesion edge (C). However, closer inspection of the PRDX2 expression revealed that there were also some PRDX2-expressing astrocytes outside the lesion, as well as some PRDX2-negative astrocytes inside the lesion. Thus, PRDX2 expression was quantified separately in different lesion areas as described in the main paper and shown in Figure 2. Note the PRDX2-positive erythrocytes in C. Since PRDX2 is highly expressed in erythrocytes, they can serve as an internal positive control. Scale bars: **A**: 200 µm, **C–B**: marked areas in **A**.

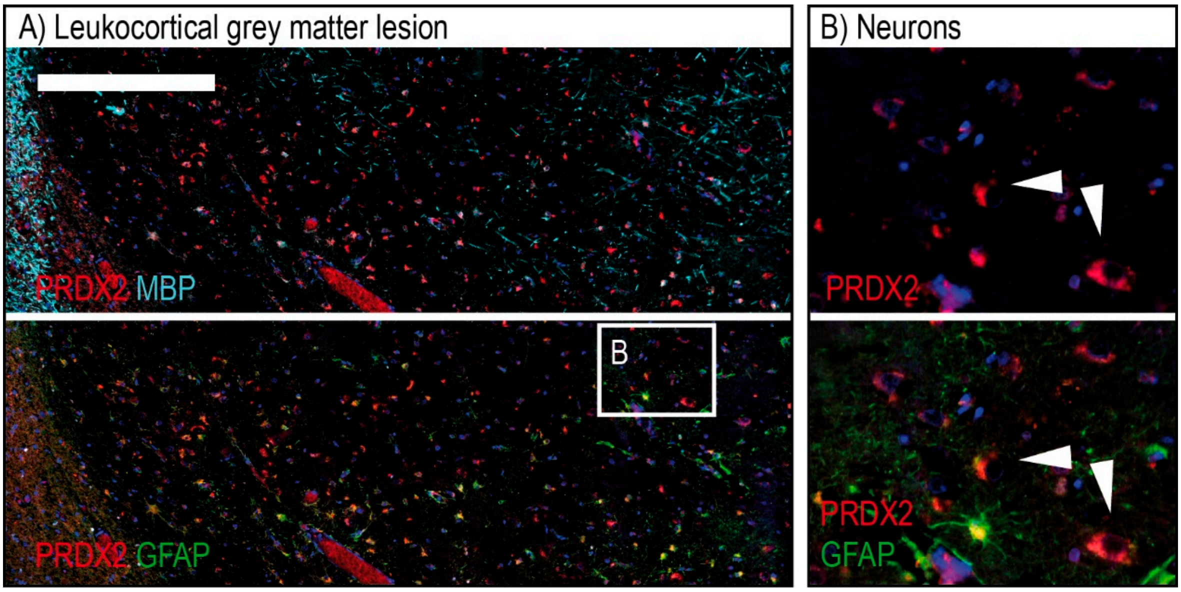

Figure S2. Expression of PRDX2 in neuronal cells in a leukocortical grey matter MS lesion. Staining for the myelin protein MBP (cyan) was used to illustrate demyelination (A). Note the sharp edge of the lesion on the left hand side and the positive MBP signal (cyan) of the myelinated adjacent white matter. Since it was difficult to establish fluorescence staining for common neuronal markers (e.g. NeuN) on the paraffin-embedded autopsy tissue, we analyzed the already existing slides stained for PRDX2 and GFAP. In the grey matter and especially in leukocortical lesions (shown in this figure) we found PRDX2-positive cells with a triangular shape which were negative for GFAP and thus most likely neurons (B, arrows). Scale bars: **A**: 500 µm, **B**: marked area in **A**.

Table S1. Sample characterization.

	MS autopsies	Control autopsies
**Number of patients**	10	7
**Number of investigated tissue samples**	17	7
**Number of tissue samples from different brain regions**		
Frontal lobe	10 (59%)	5 (71%)
Temporal lobe	3 (18%)	0 (0%)
Occipital lobe	4 (23%)	2 (29%)
**Age (mean, yrs)**	51	51
**Sex**		
Female	4	5
Male	6	2

Table S2. Antibodies and staining procedures.

Antigen/Antibody	Antibody species	Dilution	Pretreatment	Company
CD3	rat mc	1:50	citrate	ABD Serotec Puchheim Germany MCA1477
GFAP	mouse mc	1:50	-	DAKO Deutschland GmbH Hamburg, Germany M0761
KiM1P CD68 equivalent antibody	mouse mc	1:5000	citrate	Kind gift from Prof. HJ Radzun University Medical Center Göttingen, Germany
MBP	rabbit pc	1:2000	-	DAKO Deutschland GmbH Hamburg, Germany A0623
MBP	rat mc	1:200	citrate	Abcam Cambridge United Kingdom ab7349
NQO1	mouse mc	1:100	citrate	ABD Serotec Puchheim Germany MCA2880GA
PLP	mouse mc	1:500	citrate	ABD Serotec Puchheim Germany MCA839G
PRDX2	rabbit pc	1:100	citrate	Abcam Cambridge United Kingdom ab15572

Abbreviations: mc: monoclonal, pc: polyclonal
